# Meeting report: the 2020 FSHD International Research Congress

**DOI:** 10.1186/s13395-020-00253-2

**Published:** 2020-12-08

**Authors:** Michael Kyba, Robert J. Bloch, Julie Dumonceaux, Scott Q. Harper, Silvère M. van der Maarel, Francis M. Sverdrup, Kathryn R. Wagner, Baziel van Engelen, Yi-Wen Chen

**Affiliations:** 1 Department of Pediatrics, University of Minnesota, Minneapolis, MN 55104 USA; 2 Department of Physiology, University of Maryland School of Medicine, Baltimore, MD 21201 USA; 3 NIHR Biomedical Research Centre, University College London, Great Ormond Street Institute of Child Health and Great Ormond Street Hospital NHS Trust, London, WC1N 1EH UK; 4 Department of Pediatrics, The Ohio State University, Columbus, OH 43205 USA; 5 Center for Gene Therapy, The Abigail Wexner Research Institute at Nationwide Children’s Hospital, Columbus, OH 43205 USA; 6 Department of Human Genetics, Leiden University Medical Center, Leiden, The Netherlands; 7 Edward A. Doisy Department of Biochemistry and Molecular Biology, Saint Louis University, Saint Louis, MO 63104 USA; 8 Center for Genetic Muscle Disorders, Kennedy Krieger Institute, Baltimore, MD 21205 USA; 9 Departments of Neurology and Neuroscience, Johns Hopkins School of Medicine, Baltimore, MD 21205 USA; 10 Department of Neurology, Radboud University Medical Center, Nijmegen, The Netherlands; 11 Department of Genomics and Precision Medicine, The George Washington University, Washington, DC 20052 USA; 12 Center for Genetic Medicine Research, Children’s National Research Institute, Washington, DC 20010 USA

**Keywords:** Facioscapulohumeral muscular dystrophy, Muscular dystrophy, Meeting

Facioscapulohumeral muscular dystrophy (FSHD) spent many years in a wilderness of unexplained genetic mechanism while other monogenic muscular dystrophies saw rapid progress on genetic mechanism soon after their corresponding genes were discovered, with current interventions developed based on these discoveries. While it is now the consensus of the field that aberrant expression of the DUX4 transcription factor is ultimately responsible for FSHD, the linkage of such expression to muscle degeneration through a specific pathological mechanism has proven elusive, a concerning situation for many developing therapies. The 2020 Facioscapulohumeral Muscular Dystrophy (FSHD) International Research Congress, held online, June 25–26, and involving 280 registered participants from 5 continents, revealed strides to bridge this gap, as well as steps toward therapy, including the initiation of the first clinical trial specifically targeting DUX4 expression.

## Session 1: discovery research and models

Understanding which cell type DUX4 acts in, and what its pathological effects are in that cell type, benefit from investigating pathology at multiple levels, and advances highlighted in this area ranged from cell to tissue to system-wide. Major findings include the potential role of DUXA in sustaining DUX4 effects, DUX4 effects on muscle regeneration, novel circulation biomarkers, and a fish model for studying DUX4 effects.

Because DUX4 is known to impair myoblast differentiation, and has been proposed to have effects on satellite cells, Peter Zammit (Kings College, London) investigated to what extent regeneration is occurring in FSHD muscle. PAX7 and DUX4 are known to have mutually inhibitory effects on gene expression, suggesting that the expression of DUX4 in satellite cells would reduce myogenic potential. Consistent with this, myogenic and satellite cells in murine models of FSHD express DUX4. Muscle regeneration in mice carrying a DUX4-βgal reporter gene and challenged with cardiotoxin shows upregulation. PAX7+ satellite cells show similar upregulation. This suggests that DUX4 expression accompanies the activation of the myogenic program in muscle stem cells. Transcriptomic studies of regenerating healthy and FSHD muscle show that myogenic gene expression is elevated in FSHD muscles compared to controls. At the protein level, assayed by immunofluorescence, developmental myosin heavy chain was also elevated in FSHD vs controls. Most FSHD biopsies show regenerating fibers, with 0.48% of fibers in FSHD quadriceps and 1.72% of fibers in FSHD tibialis anterior muscle are regenerating. Regeneration correlates with overall severity of pathology. These results indicate that active regeneration occurs in FSHD muscle, but at low levels that are insufficient for homeostatic maintenance.

Katherine Williams (UC Irvine) has studied gene expression in nuclei of FSHD2 myoblasts and myotubes in vitro. DUX4 has been shown to spread from one nucleus to others nearby to induce target gene expression, which can persist even after DUX4 is no longer detectable. RNAseq of differentiating FSHD2 and control cell lines from myoblasts into early (day 3) myotubes identifies 54 mRNAs expressed in FSHD2 but not controls, including typical DUX4 targets as well as DUXA and LEUTX. Single nucleus analysis in myoblasts and early myotubes shows similarities between FSHD2 and control myoblasts but differences in myotubes. FSHD2 nuclei isolated from myotubes either express DUX4 or do not; they were categorized as HI or LO expressers. DUX4 target genes tend to be expressed in the HI group but not consistently, as they were also expressed at variable levels in the LO group. Their levels did not parallel levels of DUX4 in the HI group, with the exception of DUXA. Most FSHD2 myonuclei do not express DUX4, but SLC34A2 and LEUTX can be imaged at high levels in myotubes, even when DUX4 protein cannot. Differentiation for an additional 2 days (to day 5) does not significantly alter these results but shows higher levels of target gene expression. RNAseq identified ~ 1500 gene products that were elevated in expression in the HI compared to the LO group, including 6 transcription factors involved in the cell cycle. This is surprising, as nuclei in myotubes should be in G0. Suppression of expression of DUXA can suppress the expression of ZSCAN4 and LEUTX in more mature myotubes, even when DUX4 cannot be detected. This suggests that DUXA can perpetuate the abnormal gene program initiated by DUX4 expression in FSHD2.

Maria Traficante (University of Maryland Baltimore) presented preliminary evidence that serum levels of SLC34A2 are elevated in mice carrying mature FSHD human muscle. SLC34A2 is typically expressed in epithelial cells and as part of the DUX4 program in FSHD muscle. It is not found at significant levels in healthy muscle or in normal human serum. Previous work had shown that SLC34A2 could be labeled in FSHD muscle biopsies more than control biopsies and in xenografts of FSHD myogenic precursor cells (MPCs) more than control MPCs. qPCR shows higher levels of SLC34A2 mRNA in two different FSHD cell lines (15A, C6) compared to appropriate controls (15 V, A4), suggesting that its elevation is not cell-line specific. Western blots of xenografts also show elevated SLC34A2 protein. Blots of serum from mice carrying FSHD xenografts prepared with 15A MPCs also showed elevated levels of SLC34A2 protein, compared to control 15 V MPC grafts. These results suggest that SLC34A2 may be a reliable serum biomarker for FSHD.

Yuanfan (Tracy) Zhang, (Children’s Hospital, Boston) working in the Kunkel laboratory, has been examining the effects of DUX4 overexpression in zebrafish. Previous studies showed that injection of DUX4 into fish embryos results in asymmetric effects on muscle that mimic the human disease in important ways. RNAseq of injected fish show 338 genes upregulated and 10 genes downregulated, of which 55 genes show changes similar to those seen in human FSHD muscle cells. Finer regulation of DUX4 expression is achieved with a tamoxifen-inducible promoter. When expressed at low levels, DUX4 causes a milder myopathy. Screening these fish in 96-well dishes is an efficient way of identifying small molecules that can correct for the effects of DUX4. Zhang presented data to show that both herbimycin and rapamycin decrease DUX4 expression and preserve healthier muscle morphology, with reduced apoptosis and fat and fibrotic infiltration. The inducible DUX4 model in zebrafish may therefore be a good in vivo screen for drugs that suppress DUX4.

## Session 2: genetics and epigenetics

Because most cases of FSHD do not involve specific point mutations, but rather copy number alterations of a 3.3 kb macrosatellite repeat unit leading to changes in its epigenetic regulation, genetic diagnosis can be challenging in many cases. The presentations in the genetics and epigenetics section reported new and improved approaches for determining the D4Z4 repeat number and DNA methylation state. In addition, a study reporting patients carrying shortened D4Z4 array on chromosome 10 with a distal FSHD-permissive sequence strengthened the essential role of DUX4 in the disease mechanism.

Sven Bockland (BioNano Genomics) showed results from their efforts to characterize the D4Z4 repeat by optical mapping technology. Accurate sizing and chromosome assignment is possible by optical mapping, and an automated workflow and reporting for DNA diagnostics of FSHD was presented which is currently implemented for clinical testing in several centers. The technology also allows for the detection of somatic mosaicism down to 6.25% allele fraction, and future applications include the detection of D4Z4 methylation. Next, Alexander Liu (Children’s National Washington DC) shared his experience with Oxford Nanopore long read sequencing of the D4Z4 repeat. He presented a CRISPR/Cas9-based enrichment protocol resulting in 6–150-fold enrichment of the D4Z4 repeat and sequencing of the contracted and non-contracted D4Z4 repeat from both chromosome 4 alleles in FSHD1 patients. This method also allows for methylation analysis and the first data points towards uneven methylation distribution over the entire repeat with evidence for hypomethylation of the contracted allele. In the final presentation, Richard Lemmers (Leiden University Medical Center) presented two families with evidence for linkage of FSHD with chromosome 10. In both families, de novo translocations between chromosome 4- and 10-derived repeats were detected resulting in contracted repeats on chromosome 10 ending with a typical FSHD-permissive chromosome 4 sequence that allows for stable DUX4 expression. The probands of both families have a classical FSHD phenotype and express DUX4 and DUX4 target genes in their muscle cell cultures suggesting that, independent of chromosomal localization, reactivation of DUX4 in skeletal muscle causes FSHD.

In the poster session, Nicolay Zernov presented a qPCR-based approach for FSHD1 diagnostics based on DNA digestion by EcoRI, separation by pulsed field gel electrophoresis, fragmentizing according to a size standard, and using the fragments as PCR template. Darina Šikrová presented a FSHD patient with a homozygous *LRIF1* variant associated with D4Z4 hypomethylation and DUX4 expression identifying *LRIF1* as novel disease gene. Jon Thomason (University of Iowa) presented a validation study of optical mapping for the molecular diagnosis of FSHD in 40 subjects emphasizing the accuracy, robustness, preciseness, and reproducibility of this technique. Another optical mapping study by Hayk Barseghyan (Children’s National Washington DC) provided proof of principle for methylation analysis of D4Z4. Experience with DNA diagnosis of FSHD by Southern blotting was presented by Sabrina Pagnoni (Catholic University of Córdoba) representing the first molecular characterization of D4Z4 alleles and haplotypes in Latin-America. Autumn Rieken (University of Iowa) presented a retrospective analysis of CLIA laboratory testing for FSHD showing an overall positive testing rate of 42%, of which 7% is testing positive for FSHD2. Finally, Russell Buttefield (University of Utah) presented a strategy for the identification of genetic modifiers of FSHD severity in a large Utah kindred first described in the 1950’s.

## Session 3: pathology and disease mechanisms

In spite of a well-founded understanding of the genetic cause of FSHD, the field continues to struggle with understanding which cellular phenotypes and mechanisms are most relevant downstream of DUX4, and with understanding the pathological mechanism leading from DUX4 leakage from improperly silenced D4Z4 repeats to degeneration of muscle, particularly in view of the difficulty of directly detecting the DUX4 protein in muscle sections. The pathology and disease mechanisms session addressed these issues with a diverse set of talks on both molecular and tissue-level effects of DUX4 expression. While involvement of hypoxic signaling, mis-spliced RNAs, and cell death in FHSD has been reported, new findings provided insights and details on how the pathways mediate DUX4-induced cytotoxicity. In addition, the importance of expression levels and expression patterns of DUX4 was studied and reported using animal models of FSHD.

Angela Lek of the Yale School of Medicine presented results of a whole-genome CRISPR screen for knockouts that protect DUX4-expressing myoblasts from cell death. This identified a number of genes associated with hypoxia signaling. While DUX4 has been known to enhance the sensitivity of cells to oxidative stress for some time, this is the first time that the signaling activity of the pathway itself, as opposed to the potential direct pathological effects of oxidative damage, has been shown to be deleterious. The screen was followed by experiments using chemical approaches to diminish hypoxia signaling in vitro as well as in vivo using a mouse model based on FSHD human muscle xenografts, which revealed in vivo relevance of hypoxia signaling to DUX4-induced pathology at the tissue level, possibly at the level of DUX4 protein accumulation.

Amy Campbell of the University of Colorado presented work following up on the discovery that DUX4 impairs nonsense-mediated RNA decay. She showed that transcripts bearing frameshift mutations because they are not eliminated lead to immunologically detectable neo epitopes in cells expressing DUX4, particularly of factors involved in splicing. One specific factor, a truncated form of SRSF3 derived from an alternatively spliced transcript that is normally rapidly degraded, was found to be specifically deleterious to cells when overexpressed, potentially accounting in part for the DUX4 cell death phenotype.

Julie Dumonceaux of University College London found surprisingly that while caspase inhibitors failed to protect DUX4 expressing cells from death, one necroptosis inhibitor did. She tested the in vivo relevance of the necroptosis pathway by crossing the Ripk3 knockout into the background of an FSHD mouse model based on muscle-specific DUX4 expression and found a significant diminution of the pathological phenotype.

Joel Chamberlain of the University of Washington presented experiments to test direct delivery of a human FSHD D4Z4 fragment encoding DUX4 using AAV9. The construct uses the endogenous DUX4 promoter and resulted in dose-dependent pathology of skeletal muscle, including signs of fibrosis, regeneration, and fiber splitting. Interestingly, levels of DUX4 expression were almost undetectable in the lowest dose observed to present a detectable phenotype, much like the situation in humans, where DUX4 is virtually undetectable immunohistochemically in human muscle biopsy specimens.

Michael Kyba of the University of Minnesota presented work with the iDUX4pA mouse model in which DUX4 can be expressed specifically in muscle fibers when mice are treated with doxycycline (dox). Because the system is reversible with dox withdrawal, the group investigated the long-term effects of burst (single dox injection) or pulse (10 days of dox injections) expression of DUX4. On the positive side, muscle was found to recover to relatively healthy histology several months after a pulse of DUX4 expression, supporting the therapeutic potential of inhibiting DUX4. On the disconcerting side, the group found that the fibroadiopogenic progenitor compartment does not return to normal, even after several months, and proposed a model in which long-term abnormalities in these cells lead to progressive pathology, now uncoupled from DUX4 expression, raising the question of the extent to which DUX4 suppression alone would be sufficient to treat FSHD.

## Session 4: interventional strategies

This session featured presentations from several laboratories working to develop therapeutic strategies for FSHD. The main efforts focus on reduction of DUX4 transcripts directly using various strategies, including antisense oligonucleotides, CRISPR-Cas system, miRNAs, and siRNAs. In addition, strategies that modulate DUX4 expression via upstream pathways were reported, including the first clinical trial on a repurposed drug, losmapimod, that modulates DUX4 expression.

Two talks from Rika Maruyama (University of Alberta) and Yi-Wen Chen (Children’s National Hospital, George Washington University) described the development of gapmer antisense oligonucleotides modified with locked nucleic acid (LNA) or 2’-O-methoxy-ethyl (2’-MOE) bases, designed to knock down DUX4 mRNA using an RNAse H-mediated mechanism. Rika Maruyama presented the screening of the antisense oligonucleotides (AOs) in vitro, and Yi-Wen Chen reported data from in vivo experiments in uninduced FLExDUX4 mice that express very low levels of DUX4 and display mild myopathic phenotypes. Following subcutaneous AO delivery, FLExDUX4 mice showed increased grip strength and reduced fibrosis, while muscle weight was not affected. Future studies will be aimed at improving in vivo delivery to muscle, which is currently a barrier to translating oligonucleotide-based strategies for muscle diseases.

Two talks described AAV-based gene therapy strategies to inhibit DUX4 mRNA using different mechanisms in DUX4-expressing human cells and mouse models. In the first, Afrooz Rashnonejad (Nationwide Children’s Hospital, Columbus, Ohio) used a new type of CRISPR-Cas system that relied upon the RNA-targeting enzyme Cas13b, which can be directed to silence DUX4 mRNA without risk of cutting the genome. As Cas13b was too large to allow co-packaging of a guide RNA expression cassette in the same AAV genome, the first-generation system required injection of 2 AAVs, one expressing the guide RNA from a U6 promoter and a second carrying the Cas13b protein expression cassette. This system reduced DUX4 expression in vitro and in vivo, and improved DUX4-associated muscle histopathology in DUX4-expressing mice. The authors are now optimizing the vector to improve its efficiency and reduce off targets in vivo, and are also testing smaller versions of Cas13 that allow co-packaging with a gRNA in the same vector.

In the second gene therapy talk, Lindsay Wallace (Nationwide Children’s Hospital, Columbus, Ohio) presented advancements in the development of an AAV RNAi-based gene therapy for FSHD. This group has previously published several articles demonstrating efficacy of RNAi therapy in mouse models and is now optimizing the strategy for efficacy and safety for translation to clinical trial. Dr. Wallace reported new unpublished, long-term functional and histopathological improvements in TIC-DUX4 mice treated systemically with AAV9 and AAV6 vectors carrying their team’s lead sequence, called miDUX4.405. In addition, she summarized a blinded toxicology study in mice, which supported the safe use of miDUX4.405 at clinically relevant doses.

Katelyn Daman (UMass Medical School) presented a combined ex vivo and xenograft pipeline for FSHD drug development. Compounds targeting intersectional pathways in FSHD cells, or siRNAs targeting DUX4, were evaluated in vitro and in immunodeficient mouse muscles xenografted with FSHD patient myoblasts. Dr. Daman reported the identification of two promising compounds that led to decreased DUX4 target gene expression. Importantly, one compound is a repurposed drug already used in humans for another indication, thereby potentially accelerating its path to translation for efficacy testing in FSHD.

Finally, two noteworthy posters were presented by companies developing FSHD-focused technologies. Fulcrum Therapeutics presented a poster on the evaluation of p38α/β target engagement biomarkers in skeletal muscle in trials of losmapimod, which is a DUX4-reducing small molecule currently being tested in a Phase 2b (NCT04003974) study in FSHD patients. The microRNA therapeutics company miRecule presented the development of an anti-DUX4 modified RNA oligonucleotide conjugated to miRecule’s antibody delivery technology for the treatment of FSHD. The goal of this strategy is to improve oligonucleotide delivery to muscle when delivered systemically.

## Session 5: clinical studies and outcome measures

In the past couple of years, there has been increased activity in clinical research in FSHD with interventional trials, imaging studies, biomarker studies, and a large multisite natural history study. One of the most significant challenges is the slow course of the disease, which necessitates trials of long duration or large size. A key question is whether specific biomarkers can provide surrogate measures of functional efficacy and thus increase power. These studies are revealing the feasibility of clinical trials in this disease and approaches to evaluate efficacy. They have also contributed to a better understanding of FSHD

Christopher Banerji kicked off this session with a description of self-reported symptoms in the FSHD1 UK registry (*n* = 643). The authors described four clinical presentations of FSHD1: a classical presentation (74%) describing a descending myopathy, and three facial sparing phenotypes—a mild presentation (5%) with later facial and periscapular involvement, an early shoulder presentation (10%) with accelerated periscapular weakness, and an early foot presentation (9%) with accelerated foot dorsiflexor weakness. Interestingly, the authors also found that pregnancy and carrying multiple children to term was associated with slower onset of all muscle symptoms. Although this is contrary to anecdotal reports of many women affected with FSHD who feel that their pregnancy accelerates their symptoms, it is in line with other studies that have suggested a protective effect of estrogen on the development of weakness in FSHD. Peter Lunt presented scatter plots created from his own and others’ published data, providing a visual illustration of the interrelationships of various factors influencing phenotype. One of the most interesting findings was that these plots illustrate reduced methylation and earlier onset following grandmaternal-maternal versus grandpaternal-paternal transmission.

Rabi Tawil described the launch of a phase 2b trial of losmapimod in FSHD1. Losmapimod is a small molecule inhibitor of p38α/β which in preclinical studies resulted in dose-dependent reduction of DUX4 protein. In a RDBPC trial sponsored by Fulcrum Therapeutics, 76 individuals with genetically confirmed FSHD1, age 18 to 65, having a clinical severity score of 2 to 4 (Ricci scale 0–5), and a STIR+ skeletal muscle identified by MRI were randomized 1:1 to 15 mg losmapimod or placebo PO BID for 24 weeks. The primary outcome measure is change from baseline in DUX4 activity measured by quantitative polymerase chain reaction (qPCR) of a STIR+ skeletal muscle using a subset of DUX4-regulated gene transcripts. Michelle Mellion described the challenges that the COVID-19 pandemic has introduced into the conduct of clinical trials, particularly in the losmapimod trial (ReDUX4). The ReDUX4 protocol was amended to include safety monitoring through virtual visits, mobile phlebotomy, direct to patient shipment of investigational drug, and extension of the randomized controlled portion of the trial from 24 to 48 weeks to ensure capture of key assessments. Lucienne Ronco described results from a biomarker study to identify a set of stable DUX4-regulated gene transcripts that will provide a PD biomarker endpoint to measure losmapimod treatment effect. Sixteen subjects who met inclusion criteria similar to the ReDUX4 study were enrolled and underwent needle muscle biopsies of a STIR+ muscle 6 weeks apart. Using RNA-seq data from this and published studies, a panel of DUX4-related transcripts was identified.

Jeffrey Statland described the results of a phase 2 trial of ACE-083 in FSHD sponsored by Acceleron Pharma. ACE-083 is a locally delivered nonspecific myostatin inhibitor which induces increased muscle growth. This was a two part study: part 1 was dose-ranging (*N* = 37); part 2 was RDBPC for 6 months followed by a 6-month open-label period. Patients were treated with ACE-083 240 mg/muscle or placebo (1:1) injected into the tibialis anterior (TA) or biceps brachii (BB) muscles bilaterally q3 weeks (*N* = 58). The primary endpoint was increased in muscle mass of the TA or BB. ACE-083 was generally safe and well tolerated. There were mean increases in muscle volume of 13.8% (2.9) for ACE-083 versus 4.3% (2.7) for placebo (*p* = 0.01) in TA, and increases of 19.1% (2.8) for ACE-083 versus 2.7% (2.8) (*p* < 0.0001) for placebo in BB. Thus, the study met its primary endpoint. However, since there was no associated increase in function, development of ACE-083 for FSHD was terminated.

## Industry panel

The industry panel offered an opportunity for biotechnology and pharmaceutical companies to introduce themselves, their platforms, and interests, to facilitate collaborations and partnerships with the research community. This year’s panelists included Romesh Subramanian from Dyne Therapeutics, who highlighted Dyne’s delivery technology that enables targeting therapeutics to muscle. Michelle Mellion from Fulcrum Therapeutics detailed Fulcrum’s commitment to FSHD and the company’s poster and oral presentations on topics from biomarkers and clinical trial design to initiation of a phase 2b clinical trial with the p38 inhibitor losmapimod. Anthony Saleh from miRecule discussed preclinical progress with his company’s antibody-mediated muscle-targeting platform in delivering DUX4-targeting RNA therapeutics. Finally, Jane Owens from Pfizer highlighted a poster presentation of their effort to establish relevant cell assays for FSHD and went further on to discuss unanswered questions around pathophysiology of disease and development challenges that remain.

In addition to the scientific sessions and an industry panel session, the FSHD society recognized three outstanding young FSHD investigators (Angela Lek, Yale University; Karlien Mul, Radboud University Medical Center; and Sujatha Jagannathan, University of Colorado) and gave a poster award to Darina Šikrová, Leiden University Medical Center, and Kohei Hamanaka, National Institute of Neuroscience, Japan.

## Conclusions and future directions

As investigation into the molecular consequences of DUX4 expression matures, the myriad of altered pathways continues to grow. Methods to narrow down from those observed in experimental models to those relevant to human muscle degeneration will be necessary to determine which are most relevant or whether pathology is due to the combination of many perturbations.

Regarding therapies to inhibit DUX4, recent work has highlighted the potential of drugs that reduce DUX4 expression but that have systemic consequences in many other pathways, as well as strategies that are specific to DUX4, but challenging to effectively and specifically deliver to most relevant cell types in muscle. With the former currently in clinical trials, the field awaits eagerly the first results, while hoping for the development of approaches to advance the latter.

The 2020 FSHD International Research Consortium Congress brought together 280 participants from around the world to present research findings and exchange ideas. Scientific highlights include new insights in disease mechanisms, cutting-edge approaches for disease diagnosis, and ongoing pre-clinical and clinical studies of potential treatments for FSHD. The 2021 FSHD International Research Consortium is planned to be at Leiden, The Netherlands, highlighting the worldwide nature of the meeting.

## Data Availability

Not applicable.

## Abbreviations

2’-MOE: 2’-O-methoxy-ethyl
AAV: Adeno-associated virus
AO: Antisense oligonucleotide
BB: Biceps brachii
BID: Twice per day
dox: Doxycycline
FSHD: Facioscapulohumeral muscular dystrophy
LNA: Locked nucleic acid
MPC: Myogenic precursor cell
PO: Orally
qPCR: Quantitative polymerase chain reaction
RDBPC: Randomized double-blind placebo-controlled
TA: Tibialis anterior

